# Psychometric Validation of a Family Adaptation Measure for First-Episode Psychosis

**DOI:** 10.3390/healthcare14213562

**Published:** 2026-10-22

**Authors:** Wilai Napa, Nantiya Ekathikhomkit, Pennapa Unsanit, Patraporn Bhatarasakoon

**Affiliations:** 1 Ramathibodi School of Nursing, Faculty of Medicine Ramathibodi Hospital, Mahidol University, Bangkok 10400, Thailand; 2 Faculty of Nursing, Chiang Mai University, Chiang Mai 50200, Thailand

**Keywords:** family, caregiving, first-episode psychosis, adaptation, psychometric approach

## Abstract

**Background:** Contemporary tools for assessing caregiver adaptation are often developed in Western contexts, limiting their applicability to capturing authentic caregiving experiences in diverse cultural settings such as Thailand. To address this gap, this study developed and validated a psychometric scale to assess family adaptation and caregiving role engagement among relatives of individuals with first-episode psychosis in Thailand. **Method:** Using a sequential mixed-methods design, focus group interviews were initially conducted with 17 caregivers to identify key experiential dimensions. The resulting instrument was subsequently administered to 171 caregivers evaluated using exploratory factor analysis and to 172 caregivers evaluated using confirmatory factor analysis. The final 23-item scale comprises four dimensions: Consequences at the illness onset, Caregiving consequences, Psychosis recognitions, and Transportation and uncontrolled behavior challenges. **Result:** All dimensions demonstrated strong psychometric properties, with factor loadings ranging from 0.83 to 0.95, Cronbach’s alpha coefficients exceeding 0.94, and satisfactory model fit indices. **Conclusions:** Ultimately, this 23-item scale provides mental health professionals with a practical and reliable instrument to systematically identify caregiver needs, enabling tailored interventions that can improve care outcomes for individuals experiencing psychosis and their families.

## 1. Introduction

First-episode psychosis (FEP) is a serious mental health condition marked by hallucinations, delusions, and disorganised behaviour, with a mean crude incidence of 68.4 per 100,000 person-years and increased rates in Black and Asian ethnicities [1]. Evidence from FEP in the general population showed that individuals aged 16–26 accounted for 63.17% [2]. While symptomatic remission is achievable in 83% of individuals within one year, effective management of relapse risk factors remains essential [3]. Additionally, the remission rate was significantly lower for those who had a duration of four weeks or more of an untreated period and faced poor social isolation, increased unemployment, and disorganized activities [4].

Family members and caregivers play a critical role in FEP outcomes, yet face substantial challenges, including symptom recognition, emotional distress, stigma, and barriers to accessing mental health services [5,6,7]. Evidence indicates that a supportive family environment is associated with remission [8], whereas family conflict is associated with poorer psychosocial functioning [9]. Despite their importance, caregivers frequently lack preparation for their multifaceted responsibilities, which encompass information-seeking, facilitating treatment access, managing behavioural symptoms, and providing ongoing emotional support [10]. Moreover, carers faced emotional distress and psychological burden, disruption of family roles and relationships, and challenges with the accessible health care system [11].

Existing tools designed to assess caregiver adaptation have been developed in specific cultural contexts, such as the Brazilian version [12] Chinese version [13], and Japanese version [14], and may not adequately capture caregivers’ experiences in other settings, such as Thailand. Accordingly, the present study aimed to develop and psychometrically validate a culturally appropriate instrument to assess family adaptation and caregiving role engagement in the context of FEP in Thailand, enabling mental health professionals to identify caregiver needs and design targeted, culturally sensitive interventions.

### Significant Background

Research on family caregiving for individuals with first-episode psychosis (FEP) has drawn upon multiple theoretical frameworks, including the stress-appraisal framework, the McCubbin family model [15], the family resiliency model, and family-centered care, to understand caregiver adaptation and family dynamics. Collectively, these frameworks highlight that family resiliency, open communication, life balance, adaptive strategies, and a nurturing family atmosphere are critical determinants of effective caregiving and reduced caregiver stress.

The stress-appraisal framework developed by Lazarus and Folkman has been particularly influential in examining caregiver burden and perceived stress. A prominent instrument derived from this framework is the 66-item Experiences of Caregiving Inventory (ECI) [16], which assesses both negative and positive dimensions of the caregiving experience, encompassing difficult behaviours, stigma, loss, dependency, and positive relational aspects. The ECI has demonstrated strong psychometric properties across cultural settings, including the Brazilian version, for which a Cronbach’s alpha of 0.95 was reported [12], a Chinese version assessing family coping during adapting caregiving to their relatives with FEP, with a Cronbach’s α of 0.714 for the overall questionnaire and ranging from 0.57 to 0.79 for individual scales [15], another Japanese version of the Schizophrenia Caregiver Questionnaire (J-SCQ), assessing caregivers of schizophrenia, with Cronbach’s alpha coefficients of various supra-domains and subdomains of the J-SCQ being over 0.71 [14], and Thailand, where the adapted version yielded a Cronbach’s alpha of 0.78 [17].

In the Thai context, several instruments have been adapted and validated to assess caregiver experiences across diverse conditions. These include the Caregiver Burden Inventory for caregivers of individuals with dementia, the Involvement Evaluation Questionnaire and Ways of Coping Questionnaire for caregivers of individuals with major depressive disorder, and the Perceived Stress Scale, all of which have demonstrated acceptable to strong reliability [18,19,20]. Additionally, the Thai version of the Coping and Adaptation Processing Scale–Short Form, grounded in Roy’s nursing theory, has been employed to evaluate caregiver adaptation in the context of head injury management, yielding a Cronbach’s alpha of 0.87 [21].

Despite the breadth of existing research, the specific adaptation processes of caregivers managing individuals with FEP remain insufficiently understood, particularly within the Thai cultural context. Existing instruments have largely been developed and validated in other cultural settings and may not fully capture the nuanced experiences of Thai caregivers. In response, the present study developed a culturally tailored instrument to assess caregiver adaptation in the context of FEP in Thailand, with the aim of informing targeted interventions to enhance caregiver support and overall caregiving effectiveness.

## 2. Materials and Methods

**Design:** A mixed-methods approach was employed to explore family role engagement, integrating both qualitative and quantitative methods. In the first phase, interview questions were formulated based on the concept of family caregiving for relatives with first-episode psychosis [22]. Focus group interviews were conducted to explore various aspects of family caregiving and adaptation, yielding items that captured all dimensions of family adaptation. Subsequently, in the second phase, a quantitative approach was employed to develop and validate items for psychometric testing, ensuring they accurately reflected family adaptation processes.

**Sampling:** In the first phase of the qualitative study, purposive sampling was used to select participants who were primary or secondary caregivers. Selection criteria included being over 18 years of age and providing care for relatives with first-episode psychosis for 20–25 h per week since illness onset, an additional cognitive assessment (Montreal Cognitive Assessment-Thai Version) for participants who were aged 60 years and over; if the score was below 25, they were excluded. According to the patients diagnosed with their first episode of psychosis of schizophrenia spectrum disorders, excluding schizoaffective and substance-induced psychosis, and presenting illness for two to five years. Based on these sampling criteria, nurses in the psychiatric hospital’s outpatient department approached 17 potential participants and invited them to take part in the study. Subsequently, the researchers explained the study to participants and obtained informed consent following approval from the ethics committee of the X Hospital Board. Focus group interviews were conducted with 5–10 individuals per group, each lasting 1–1.5 h. Questions explored participants’ perspectives on caregiving and engagement in the caregiver role, including: “What was your life like when your relative was first diagnosed with psychosis?” “How do you feel about psychosis?” “How do you care for relatives with first-episode psychosis?” and “Do you face any difficulties in assuming the caregiver role, and how do you manage that role?”

In the second phase of the quantitative method, nurses at psychiatric hospitals selected participants who met the purposive criteria and were willing to participate. Nurses recruited participants from four psychiatric hospitals: two in northeastern Thailand and two in the southern region. A total of 343 participants from these facilities provided informed consent and agreed to participate. According to the 1:10 ratio, the sample size (343) was sufficient to conduct CFA [23]. Participants completed self-report ratings using the Family Adaptation to Caregiving Role Instrument (FACRI), a 29-item instrument developed from phase I.

**Data Collection:** Phase I: Three focus groups were conducted, with 5, 6, and 6 participants, respectively, and all sessions were audio-recorded. An experienced transcriber transcribed the recordings verbatim, and researchers reviewed all transcripts to ensure completeness and accuracy. Transcripts were prepared for analysis following standard procedures. From the focus group interviews, an initial set of 35 family adaptation items was generated. This preliminary version of the FACRI was tested with 30 primary caregivers of individuals experiencing first-episode psychosis.

Phase II: Nurses at psychiatric hospitals selected participants according to purposive criteria and obtained informed consent. A total of 343 participants from four psychiatric hospitals completed the 29-item FACRI using a five-point rating scale: 1 = strongly disagree, 2 = disagree, 3 = partially agree, 4 = agree, and 5 = strongly agree.

**Data Analysis:** Phase I: Qualitative data from three focus groups were analyzed using thematic analysis [22]. Initially, all transcripts were thoroughly reviewed to gain a comprehensive understanding of participants’ responses. Primary coding involved line-by-line coding of all words or phrases that directly addressed the research questions and objectives. For example, when a participant stated, “I was shocked at that time because my son was doing weird things, and he was not the same person,” the phrases “was shocked” and “was not the same person” were highlighted. Subsequently, all coded text was compared to identify similarities and aligned with the conceptual framework of family caregiving for relatives with first-episode psychosis [24]. Similar words and phrases were then categorized into themes. All themes and categories were discussed among researchers, with discrepancies resolved through consensus.

**Phase II: Instrument Refinement and Psychometric Validation**

The development and psychometric evaluation of the FACRI questionnaire were conducted in two sequential stages: preliminary item screening and formal construct validation.

**Stage 1: Preliminary Screening and Pilot Testing (*****n*** **= 30)**

Qualitative findings were initially operationalized into a draft 35-item questionnaire. Content validity was evaluated by three psychiatric nursing experts, achieving an Item-Level Content Validity Index (I-CVI) of 0.88. The 35-item draft was subsequently pilot-tested with 30 caregivers to evaluate item clarity, feasibility, and preliminary performance. During this pilot phase, preliminary item analysis and exploratory factor analysis (EFA) were conducted to identify redundant or non-performing items. Based on item-total correlations, extreme cross-loadings, and expert feedback, 6 items were removed, resulting in a refined 29-item version with high internal consistency (*α* = 0.87).

Responses for all items were scored on a 5-point Likert scale ranging from 1 (*strongly disagree*) to 5 (*strongly agree*).

**Stage 2: Main Psychometric Testing and Sample Splitting (*****N*** **= 343)**

The 29-item version was administered to the main validation sample of 343 caregivers. To rigorously evaluate construct validity and avoid sample overlap bias, the total dataset was randomly partitioned into two independent subsamples (n_1_ = 171 and n_2_ = 172).

• **Formal Exploratory Factor Analysis (*****n*****_1_ = 171):** An EFA using Principal Axis Factoring (PAF) with Direct Oblimin rotation was performed on the first subsample to determine the underlying factor structure. Six items were removed due to significant cross-loadings (>0.30), yielding a final 23-item, 4-factor structure that accounted for 68.71% of the total cumulative variance.
• **Confirmatory Factor Analysis (*****n*****_2_ = 172):** The second subsample was utilized for Confirmatory Factor Analysis (CFA) to cross-validate and confirm the construct validity and model fit of the newly established 23-item, 4-factor structure.

## 3. Results

The instrument’s reliability and construct validity (exploratory factor analysis [EFA] and confirmatory factor analysis [CFA]) were assessed in 343 caregivers aged 18 to 78 years (mean = 46.29 years), with the relatives’ ages ranging from 15 to 35 years, and a duration of illness of two to five years. Among participants, 254 (74.05%) were primary caregivers and 89 (25.95%) were secondary caregivers. This distribution reflects diverse familial relationships and highlights mothers’ significant caregiving role. Primary caregivers comprised mothers (n = 106; 30.90%), relatives (n = 91; 26.53%), cousins (n = 67; 19.53%), fathers (n = 43; 12.54%), husbands (n = 20; 5.83%), and wives (n = 16; 4.66%). The gender distribution was 203 (59.18%) females and 140 (40.82%) males, with greater female participation consistent with women’s predominant caregiving roles.

Regarding care duration, 202 caregivers (58.89%) provided care for 2–5 years, while 141 (41.11%) provided care for 6 months to 2 years. Most caregivers had extensive experience (2–5 years), ensuring that instrument testing was conducted by substantially experienced individuals, which was crucial for assessing reliability and validity over time. Living arrangements showed 233 caregivers (67.93%) lived with care recipients daily, 78 (22.74%) lived together 2–5 days weekly, and 21 (6.12%) lived together only on weekends. The majority’s daily cohabitation suggests that the instrument captures experiences of those deeply involved in day-to-day caregiving, highlighting varying levels of involvement and enhancing instrument robustness across diverse caregiving situations.

### 3.1. The Instrument’s Construct Validity and Reliability

**Exploratory Factor Analysis (EFA) Results**.

#### 3.1.1. Data Suitability and Diagnostic Checks

Prior to factor extraction, several diagnostic tests were conducted on the 23 items to evaluate dataset suitability for factor analysis (*N* = 171). The sample-to-item ratio was 7.43:1, meeting the recommended minimum standard of 5:1 [25,26]. The Kaiser–Meyer–Olkin (KMO) measure of sampling adequacy was 0.91, categorized as “marvelous” [27] and well above the recommended cutoff of 0.60. Bartlett’s Test of Sphericity was statistically significant (*χ*^2^ = 3696.19, *df* = 253, *p* < 0.001), indicating that the correlation matrix was not an identity matrix and possessed sufficient common variance. Diagonal values of the anti-image correlation matrix (Measures of Sampling Adequacy; MSA) for all individual items ranged from 0.82 to 0.96, exceeding the 0.50 threshold. Additionally, the correlation matrix determinant was 1.150 × 10^−10^, confirming the absence of extreme multicollinearity (*r* > 0.90). These diagnostics confirmed that the dataset was highly appropriate for factor extraction.

#### 3.1.2. Factor Extraction and Variance Explained

An initial Exploratory Factor Analysis (EFA) was conducted on all 29 items. Based on standard item-retention criteria, six items (9, 16, 21, 26, 27, and 28) exhibiting substantial cross-loadings (>0.30 across multiple factors) were systematically eliminated. A final EFA was re-run on the remaining 23 items using Principal Axis Factoring (PAF) with Direct Oblimin rotation on a sample of 171 respondents. PAF was chosen to isolate common variance, and Oblimin rotation was applied, given the theoretical and empirical correlations among factors.

The final four-factor solution achieved a clean simple structure, extracting four distinct factors with Eigenvalues exceeding 1.0. Together, these four factors accounted for 68.71% of the total cumulative common variance. Specifically, Factors 1, 2, 3, and 4 explained 48.16%, 12.32%, 4.21%, and 4.02% of the variance, respectively. Item communalities (*h*^2^) ranged from 0.46 to 0.88, well above the recommended 0.30 threshold [28]. Factor loadings from the Pattern Matrix, communalities, Eigenvalues, and variance explained are detailed in Table 1.

#### 3.1.3. Internal Consistency Reliability

Following the factor extraction, the internal consistency reliability of the refined 23-item FACRI scale and its four subscales was evaluated using Cronbach’s alpha coefficient. As shown in Table 1, the overall 23-item scale demonstrated excellent internal consistency, with a total Cronbach’s alpha of 0.95.

Furthermore, all four extracted factors exhibited high internal consistency reliability exceeding the recommended threshold of 0.70: Factor 1 (*α* = 0.93; 6 items), Factor 2 (*α* = 0.89; 6 items), Factor 3 (*α* = 0.97; 4 items), and Factor 4 (*α* = 0.92; 7 items). Corrected item-total correlations for all individual items within their respective factors were well above 0.30, confirming robust item homogeneity and construct reliability across all dimensions.”

**Confirmatory Factor Analysis (CFA) Results**

A two-stage Confirmatory Factor Analysis (CFA) using LISREL 12.4.5 was conducted to evaluate the measurement model and validate the construct’s structural integrity. Maximum Likelihood (ML) estimation was employed using the covariance matrix derived from a sample of 172 participants.

#### 3.1.4. Initial First-Order Confirmatory Factor Analysis

To evaluate the construct validity and internal structure of the measurement scale, an initial First-Order Confirmatory Factor Analysis (CFA) was conducted on the 23 observed indicators across four latent factors. The preliminary baseline model demonstrated a sub-optimal fit to the empirical data (*χ*^2^ = 679.764, *df* = 224, *p* < 0.001, *χ*^2^/*df* = 3.035, CFI = 0.868, TLI = 0.851, SRMR = 0.0650, RMSEA = 0.109, 90% CI [0.100,0.119]) [see Table 2]. Although all 23 items exhibited statistically significant standardized factor loadings (*λ* = 0.48 to 1.00, *p* < 0.001), confirming satisfactory item-level validity, the primary incremental fit indices (CFI and TLI) fell below the recommended cutoff threshold of 0.90.

#### 3.1.5. Model Respecification (Modification)

The initial first-order CFA model (23 observed items across 4 latent factors) exhibited sub-optimal fit to the empirical data. Guided by Modification Indices (MI) and substantive theoretical justification, model respecification was performed by sequentially freeing three within-subscale error covariances to account for item-level semantic redundancy: *F*11–*F*12 (*θ_δ_* = 0.17), *F*16–*F*17 (*θ_δ_* = 0.25), and *F*319–*F*320 (*θ_δ_* = 0.29). These adjustments significantly improved overall model fit (Δ*χ*^2^ = 179.50, Δ*df* = 3, *p* < 0.001), yielding satisfactory fit indices ($\chi^{2}=500.27,$  df=221,   p<0.001;  $\frac{\chi^{2}}{\mathrm{df}}=2.26;$  CFI=0.919;  NNFI/TLI=0.0908;  IFI=0.920;  SRMR=0.0584;  RMSEA=0.0860,  $90\%\;\mathrm{CI}\;\left[0.0760,0.0960\right]$; see Table 2. All standardized factor loadings remained statistically significant (*p* < 0.001), ranging from 0.48 to 1.00, and confirming robust first-order construct validity. Furthermore, substantial inter-factor correlations among Factor 1, Factor 3, and Factor 4 (*r* = 0.72 to 0.88) provided strong empirical justification for evaluating a higher-order factor structure.

#### 3.1.6. Second-Order Confirmatory Factor Analysis

**Figure 1:**
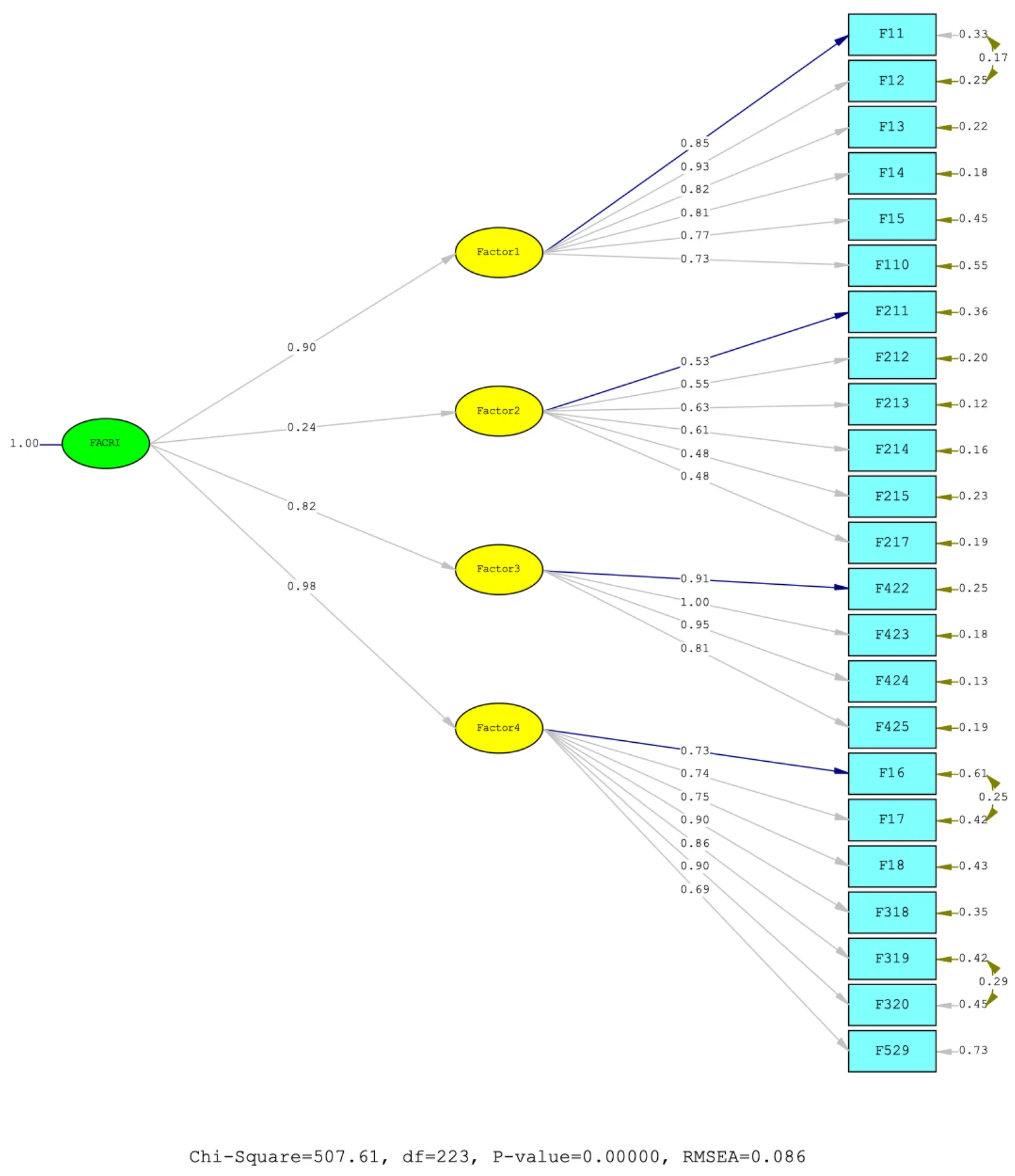
Second-order Confirmatory factor analysis of the FACRI.

Consequently, a Second-Order CFA was executed to test whether a single overarching latent construct (FACRI) accounts for the covariation among the four first-order dimensions. The second-order model demonstrated an excellent fit, virtually identical to the modified first-order baseline (*χ*^2^ = 507.614, *df* = 223, *p* < 0.001, *χ*^2^/*df* = 2.276, CFI = 0.918, TLI = 0.907, IFI = 0.919, SRMR = 0.0629, RMSEA = 0.0864, 90% CI [0.0765,0.0963]) [see Table 2 and Figure 1]. The Target Coefficient (*T*-Index) was calculated at 0.9855 (98.55%), confirming that the higher-order construct accounted for 98.55% of the first-order covariance structure.

#### 3.1.7. Factor Loadings & Construct Evaluation

As presented in Table 3, standardized second-order loadings (*γ*) were high for Factor 4 (*γ* = 0.98, *p* < 0.001), Factor 1 (*γ* = 0.90, *p* < 0.001), and Factor 3 (*γ* = 0.82, *p* < 0.001), with Factor 2 demonstrating a moderate contribution (*γ* = 0.24, *p* < 0.001). All first-order standardized loadings remained robust (*λ* = 0.48 to 1.00, *p* < 0.001). Taken together, these findings confirm the instrument’s hierarchical construct validity, supporting its dual application for reporting both subscale-specific scores and an overall composite score.

### 3.2. Internal Consistency

Internal consistency analysis (Cronbach’s alpha coefficient) for each subscale and the overall scale is presented in Table 4. Overall scale Cronbach’s alpha was 0.94, indicating excellent internal consistency. Each dimension’s Cronbach’s alpha exceeded 0.60. High alpha values (approaching or exceeding 0.95) suggest consistent measurement of the same underlying construct, while dimensional alphas > 0.60 indicate reliable subscales. These findings demonstrate that the four dimensions and 23 items of the revised Family Adaptation to Caregiving Role Instrument (FACRI) are reliable and valid measures of family adaptation in FEP caregivers. The instrument demonstrates strong internal consistency and adequate data fit, establishing it as a robust tool for assessing family adaptation and role engagement in this context.

## 4. Discussions

### 4.1. Study Objectives and Validation

This study aimed to develop a psychometric test for the FACRI to assess family adaptation and engagement in caregiving roles for relatives with first-episode psychosis among Thai caregivers. The FACRI was statistically validated using EFA and CFA. The model demonstrated that 23 items across four dimensions provided an excellent fit for explaining family adaptation to caregiving roles. The overall scale achieved a Cronbach’s alpha coefficient of 0.94, with each dimension exceeding 0.60, indicating high internal consistency. The family adaptation model comprised four factors: (1) Consequences at the illness onset, (2) Caregiving consequences, (3) Psychosis recognition, and (4) Transportation and uncontrolled behavior challenges. The operational definition suggests that families, as units, adapt to caregiving roles by managing consequences at the onset of disease, delivering proper care for relatives, recognizing psychotic symptoms, addressing transportation challenges, and managing behaviors during care provision.

### 4.2. Methodological Strengths and Validation Process

The FACRI family adaptation model evaluation began with qualitative caregiver interviews, which were analyzed using content analysis [22]. This qualitative phase developed a comprehensive understanding of caregiving experiences, informing FACRI item formulation. Expert validation through the Content Validity Index (CVI) ensured item relevance and clarity. EFA and CFA validated the FACRI, confirming effective capture of caregiver adaptation when caring for individuals with FEP. The iterative process of item development, expert review, and statistical validation underscores the instrument’s robustness.

### 4.3. Comparison with Existing Instruments

Psychosis caregiving experts evaluated the explanatory quality of the items using CVI. FACRI items underwent theoretical validation by experts, with EFA and CFA applied for validation. Additionally, existing adaptation tools were reviewed [16,18,21,22] to enable comparisons during item formulation. This comparative analysis ensured the FACRI incorporated best practices and addressed previous instrument gaps, enhancing comprehensiveness.

### 4.4. Exclusion of Six Items

The sixth dimension initially addressed family beliefs attributing psychosis to superstitious powers or substance abuse. EFA results revealed this dimension failed to meet criteria, with Cronbach’s alpha below 0.60. Families with low socioeconomic support tended to attribute psychosis to social and supernatural factors, seeking the assistance of a faith healer. Conversely, families with high socioeconomic support held opposite beliefs [17]. Furthermore, families carefully investigated illness causation, seeking explanations for their relatives’ initial psychosis. Subsequently, families recognized the importance of assuming caregiving roles for individuals with psychosis [9]. This suggests that despite differing beliefs, families provided appropriate treatment and care. Therefore, the sixth dimension was excluded as it did not reflect caregiver engagement.

## 5. Conclusions

The FACRI provides a reliable and valid measure of family adaptation and role engagement in caregiving for relatives with first-episode psychosis. The instrument’s strong psychometric properties establish it as a dependable tool for evaluating family adaptation, ensuring effective capture and understanding of caregivers’ experiences. This study underscores the importance of understanding caregiving roles, developing caregiving skills, and supporting caregivers throughout their challenging journey.

## 6. Clinical Practice Implications

The findings emphasize families’ holistic adaptation transition to caregiving roles, from initial mental illness onset to full engagement as primary caregivers. The FACRI was systematically developed and tested, achieving good model fit. For mental health professionals, the FACRI can assess family adaptation and engagement, facilitating intervention development to support families in adjusting to caregiving roles and acquiring optimal caregiving skills for individuals with first-episode psychosis. By understanding these dynamics, mental health professionals can better support caregivers, address their specific needs, and promote effective caregiving strategies.

## Figures and Tables

**Table 1:** Final Exploratory Factor Analysis Results of the FACRI Using Principal Axis Factoring and Direct Oblimin Rotation (N = 171).

Item	Statement	Communality (h^2^)	Pattern Loading			
			Factor 1	Factor 2	Factor 3	Factor 4
3 (F13)	Family members feel afraid and anxious when there is a member with psychosis in the family.	0.88	1.02			
1 (F11)	This illness makes the patient dependent on others	0.81	0.85			
2 (F12)	Family members get stressed out when there is a member with psychosis in the family.	0.88	0.85			
4 (F14)	Family members feel sorrowful and distressed when there is a member with psychosis in the family.	0.77	0.75			
5 (F15)	Family members have difficulty preventing the patient from wandering.	0.53	0.49			
10 (F110)	Family members have sleep problems when there is a member with psychosis in the family.	0.53	0.43			
12 (F212)	Family members closely monitor for side effects from psychiatric medications.	0.66		0.83		
11 (F211)	Family members keep an eye on the patient’s changing symptoms, such as irritability, stress, withdrawal, insomnia, and yelling.	0.61		0.80		
13 (F213)	Family members adjust their communication and care for the patient.	0.63		0.78		
14 (F214)	Family members help take care of the sick as much as they can.	0.70		0.75		
15 (F215)	Family members help ensure that the patient takes all the medicine.	0.56		0.71		
17 (F217)	Relatives or neighbors help keep an eye on the patient and stay aware of their condition.	0.46		0.68		
24 (F424)	The family members notice that the patient’s behavior has obviously changed, such as pacing, boasting, cursing, insomnia, paranoia, isolation, speaking, or laughing alone.	0.88			0.96	
23 (F423)	The family members notice that the patient has presented peculiar thoughts different from before.	0.88			0.88	
22 (F422)	Family members notice that the patient’s behavior has changed, either becoming a different person or exhibiting different behaviors.	0.86			0.86	
25 (F425)	The family members notice that the patient’s mood has rapidly changed, different from before.	0.744			0.76	
7 (F17)	Family members cannot predict when the symptoms will recur.	0.72				0.88
8 (F18)	Family members have difficulties in preventing the patient from smoking and drinking alcohol.	0.71				0.82
6 (F16)	Family members have difficulties in managing the patient’s medication intake.	0.71				0.78
29 (F529)	The family members face difficulties in preventing the patient from using substances.	0.57				0.69
18 (F318)	The family is inconvenienced in transporting patients to the hospital.	0.57				0.44
19 (F319)	The family has to hire vehicles to transport the patient.	0.59				0.43
20 (F320)	The family has transportation expenses.	0.56				0.42
Eigenvalue			11.08	2.84	0.97	0.93
% Variance			48.16	12.32	4.21	4.02
Cumulative %			48.16	60.48	64.69	68.71

**Table 2:** Model Fit Comparison of confirmatory factor analysis of the FACRI (n = 172).

Models	*χ* ^2^	df	*χ*^2^/df	CFI	TLI/NNFI	SRMR	RMSEA (90% CI)
1. Initial First-Order	679.764	224	3.035	0.868	0.851	0.0650	0.109 (0.100–0.119)
2. Modified First-Order	500.268	221	2.264	0.919	0.908	0.0584	0.0860 (0.076–0.096)
3. Final Second-Order	507.614	223	2.276	0.918	0.907	0.0629	0.0864 (0.076–0.096)
**Threshold**	-	-	<3.00	≥0.90	≥0.90	<0.08	<0.08 (Marginally acceptable <0.10)

Threshold: the values of standard statistic cutoffs to determine model fits.

**Table 3:** Standardized Second-Order and First-Order Factor Loadings for the Final FACRI Model (N = 172).

Construct/Indicator	Standardized Loading (γ/λ)	*p*-Value	Residual Variance (ψ/δ)	*R*^2^ (SMC)
**Second-Order Factor: FACRI**				
→Factor 1	0.90	<0.001	0.17	0.81
→Factor 2	0.24	<0.001	0.90	0.06
→Factor 3	0.82	<0.001	0.25	0.67
→Factor 4	0.98	<0.001	0.29	0.96
**First-Order Factor 1**				
→Item F11	0.85	<0.001	0.33	0.72
→Item F12	0.93	<0.001	0.25	0.86
→Item F13	0.82	<0.001	0.22	0.67
→Item F14	0.81	<0.001	0.18	0.66
→Item F15	0.77	<0.001	0.45	0.59
→Item F110	0.73	<0.001	0.55	0.53
**First-Order Factor 2**				
→Item F211	0.53	<0.001	0.36	0.28
→Item F212	0.55	<0.001	0.20	0.30
→Item F213	0.63	<0.001	0.12	0.40
→Item F214	0.61	<0.001	0.16	0.37
→Item F215	0.48	<0.001	0.23	0.23
→Item F217	0.48	<0.001	0.19	0.23
**First-Order Factor 3**				
→Item F422	0.91	<0.001	0.25	0.83
→Item F423	1.00	<0.001	0.18	1.00
→Item F424	0.95	<0.001	0.13	0.90
→Item F425	0.81	<0.001	0.19	0.66
**First-Order Factor 4**				
→Item F16	0.73	<0.001	0.61	0.53
→Item F17	0.74	<0.001	0.42	0.55
→Item F18	0.75	<0.001	0.43	0.56
→Item F318	0.90	<0.001	0.35	0.81
→Item F319	0.86	<0.001	0.42	0.74
→Item F320	0.90	<0.001	0.45	0.81
→Item F529	0.69	<0.001	0.73	0.48

Note. *γ* = Standardized second-order factor loading; *λ* = Standardized first-order factor loading; *R*^2^ = Squared Multiple Correlation (Variance explained); *ψ*/*δ* = Residual/Error variances. All loadings are statistically significant at *p* < 0.001. Correlated error terms specified during first-order model respecification: *F*11 ↔ *F*12 (*θ_δ_* = 0.17), *F*16 ↔ *F*17 (*θ_δ_* = 0.25), and *F*319 ↔ *F*320 (*θ_δ_* = 0.29).

**Table 4:** Internal consistency reliability of the FACRI (n = 172).

Factor	No. of Items	Corrected Item-Total Correlation	Cronbach’s Alpha Coefficient
Factor 1: Consequences at the illness onset	6	0.90–0.93	0.93
Factor 2: Caregiving consequences	6	0.86–0.89	0.89
Factor 3: Psychosis recognitions	4	0.91–0.95	0.95
Factor 4: Transportation and uncontrolled behaviors challenges	7	0.88–0.91	0.91

## Data Availability

The original contributions presented in this study are included in the article/Supplementary Material. Further inquiries can be directed to the corresponding author.

## Supplementary Materials

The following supporting information can be downloaded at: https://www.mdpi.com/article/10.3390/healthcare14213562/s1, Table S1. The 1st and 2nd round of exploratory factor analysis of the FACFEP (n = 30 and n = 343); Table S2. confirmatory factor analysis of the FACFEP (n = 343); Table S3. Internal consistency reliability of the FACFEP (n = 343).
