# Supplementary material for: Psychometric Validation of a Family Adaptation Measure for First-Episode Psychosis
Source: Healthcare (Basel). 2026 Oct 22;14(21):3562. doi: 10.3390/healthcare14213562 (PMC13763195; doi:10.3390/healthcare14213562)
Supplement: Supplementary file 1 [file healthcare-14-03562-s001.zip › healthcare-4582374-supplementary.pdf]

**Table S1.** The 1st and 2nd round of exploratory factor analysis of the FACFEP (n=30 and n = 343)

| Item | Statement | Factor loading | | Communalities ($h^2$) | |
|---|---|---|---|---|---|
| **Factor 1: Family consequences at the onset of disease**<br>Eigenvalue = 8.25; percent of total variance =20.12% (n=30)<br>Eigenvalue = 10.76; percent of total variance =42.58% (n=343) | | n=30 | n=343 | n=30 | n=343 |
| Family 1 | This illness makes the patient dependent on others | 0.82 | 0.99 | 0.86 | 0.80 |
| Family 2 | Family members get stressed out when there is a member with psychosis in the family. | 0.77 | 0.96 | 0.88 | 0.85 |
| Family 3 | Family members feel afraid and anxious when there is a member with psychosis in the family. | 0.76 | 0.86 | 0.85 | 0.81 |
| Family 5 | Family members feel sorrowful and distressed when there is a member with psychosis in the family. | 0.70 | 0.88 | 0.87 | 0.87 |
| Family 6 | Family members have difficulty preventing the patient from wandering. | 0.68 | 0.76 | 0.92 | 0.90 |
| Family 7 | Family members have difficulties in managing the patient's medication intake. | 0.64 | 0.75 | 0.90 | 0.92 |
| Family 8 | Family members cannot predict when the symptoms will recur. | 0.63 | 0.72 | 0.72 | 0.80 |
| Family 9 | Family members have difficulties in preventing the patient from smoking and drinking alcohol. | 0.61 | 0.68 | 0.89 | 0.88 |
| Family 9 | Family members have difficulties in preventing the patient from consuming Coca- Cola, coffee, and energy drinks. | 0.57 | 0.67 | 0.86 | 0.85 |
| Family 10 | Family members have sleep problems when there is a member with psychosis in the family. | 0.53 | 0.84 | 0.83 | 0.84 |
| Family 11 | The family members receive encouragement from others outside the family when there is a member with a psychiatric disorder. | 0.72 | 0.66 | 0.80 | 0.82 |
| Family 12 | The family members face difficulties in preventing the patient from using substances. | 0.52 | 0.65 | 0.91 | 0.89 |
| **Factor 2: Caregiving and its consequences**<br>Eigenvalue = 6.12; percent of total variance =14.19% (n=30)<br>Eigenvalue = 9.15; percent of total variance =18.26% (n=343) | | | | | |
| Care 2 | Family members keep an eye on the patient's changing symptoms, such as irritability, stress, withdrawal, insomnia, and yelling. | 0.91 | 0.84 | 0.88 | 0.87 |
| Care 7 | Family members closely monitor for side effects from psychiatric medications. | 0.82 | 0.83 | 0.92 | 0.91 |
| Care 8 | Family members adjust their communication and care for the patient. | 0.80 | 0.82 | 0.80 | 0.83 |
| Care 9 | Family members help take care of the sick as much as they can. | 0.79 | 0.78 | 0.85 | 0.87 |
| Care 10 | Family members help ensure that the patient takes all the medicine. | 0.76 | 0.75 | 0.83 | 0.85 |
| Care 11 | Family members encourage each other when a psychiatric patient is in the home. | 0.68 | 0.77 | 0.90 | 0.92 |
| Care 12 | Relatives or neighbors help keep an eye on the patient and stay aware of their condition. | 0.51 | 0.70 | 0.87 | 0.90 |
| Care 13 | Family members feel tired when caring for the patient during daily activities. | 0.68 | 0.67 | 0.87 | 0.89 |

| Item | Statement | Factor loading | | Communalities ($h^2$) | |
|---|---|---|---|---|---|
| **Factor 3: First recognize psychosis** | | | | | |
| Eigenvalue = 2.94; percent of total variance =7.17% (n=30) | | | | | |
| Eigenvalue = 7.62; percent of total variance =4.90% (n=343) | | | | | |
| Frecog 1 | Family members notice that the patient's behavior has changed, either becoming a different person or exhibiting different behaviors. | 0.84 | 0.89 | 0.86 | 0.84 |
| Frecog 2 | The family members notice that the patient's behavior has obviously changed, such as pacing, boasting, cursing, insomnia, paranoia, isolation, speaking, or laughing alone. | 0.77 | 0.86 | 0.80 | 0.81 |
| Frecog 3 | The family members notice that the patient has presented peculiar thoughts different from before. | 0.69 | 0.69 | 0.86 | 0.84 |
| Frecog 4 | The family members notice that the patient's mood has rapidly changed, different from before. | 0.66 | 0.67 | 0.86 | 0.84 |
| **Factor 4: Managing for patient getting treatment** | | | | | |
| Eigenvalue = 4.08; percent of total variance =9.95% (n=30) | | | | | |
| Eigenvalue = 8.46; percent of total variance =5.88% (n=343) | | | | | |
| Manage 1 | The family is inconvenienced in transporting patients to the hospital. | 0.90 | 0.94 | 0.96 | 0.86 |
| Manage 2 | The family has to hire vehicles to transport the patient. | 0.84 | 0.87 | 0.90 | 0.87 |
| Manage 3 | The family has transportation expenses. | 0.83 | 0.87 | 0.86 | 0.75 |
| Manage 4 | The family has difficulty requesting transportation from police officers, medical personnel, or volunteer rescue workers. | 0.64 | 0.83 | 0.77 | 0.72 |

**Table S2.** confirmatory factor analysis of the FACFEP (n=343)

| Latent variable: Family adaptive to caregiving role | Observe variables | Factor loading *b* (se) | SE | Determinant coefficient ($R^2$) |
|---|---|---|---|---|
| Factor 1: Family consequences at the onset of disease | 12 | 0.95 | 0.05 | 0.98 |
| Factor 2: Caregiving and its consequences | 8 | 0.86 | 0.03 | 0.90 |
| Factor 3: First recognize psychosis | 4 | 0.85 | 0.05 | 0.85 |
| Factor 4: Managing for patient getting treatment | 4 | 0.83 | 0.04 | 0.83 |
| Chi-Square = 1461.45, df = 346, p = .000, | | | | |
| CFI = 0.96, AGFI = 0.99, GFI = 0.95, NFI = 0.92, SRMR = 0.06, RMSEA = 0.047 | | | | |

Footnote: SE = Standard Error

CFI = Comparative Fit Index, a value equal to or over .90 is considered acceptable

NFI = Normed Fit Index, a value equal to or over .90 is considered acceptable

GFI = Goodness of Fit Index, a value equal to or over .95 is considered acceptable

AGFI = Adjusted Goodness of Fit Index, a value equal to or over .95 is considered acceptable

SRMR = Standardized Root Mean Residual, a value less than .08 is considered acceptable

RMSEA = Root Mean Square Error of Approximation, a value less than .06 is considered acceptable

**Table S3.** Internal consistency reliability of the FACFEP (n=343)

| Factor | No. of items | Corrected item-total correlation | Cronbach's alpha coefficient |
|---|---|---|---|
| Factor 1: Family consequences at the onset of disease | 12 | 0.80 – 0.93 | 0.94 |
| Factor 2: Caregiving and its consequences | 8 | 0.74 – 0.85 | 0.87 |
| Factor 3: First recognize psychosis | 4 | 0.86 – 0.91 | 0.92 |
| Factor 4: Managing for patient getting treatment | 4 | 0.85 – 0.91 | 0.95 |
